# Usefulness of Prognostic Stage in High-Risk Early Luminal Breast Cancer

**DOI:** 10.31662/jmaj.2025-0485

**Published:** 2026-07-10

**Authors:** Mio Adachi, Toshiyuki Ishiba, Takashi Kuwayama, Tomoyuki Aruga

**Affiliations:** 1 Department of Breast Surgery, Institute of Science Tokyo, Tokyo, Japan; 2 Department of Surgery (Breast), Tokyo Metropolitan Cancer and Infectious Disease Center Komagome Hospital, Tokyo, Japan

**Keywords:** MonarchE trial, prognostic stage, high-risk luminal breast cancer

## Abstract

**Introduction:**

Breast cancer remains the most prevalent malignancy among women worldwide, with prognostic outcomes and therapeutic responses differing considerably across subtypes. The conventional staging framework developed by the Union for International Cancer Control (UICC), based on the Tumor, Node, Metastasis classification, reflects anatomical stage (AS) but overlooks biological characteristics. To address this limitation, the American Joint Committee on Cancer (AJCC) introduced the Prognostic Stage (PS) in its 8th edition, which integrates biomarkers including estrogen receptor (ER), progesterone receptor (PgR), human epidermal growth factor receptor 2 (HER2), and histological grade (HG). This study aimed to assess and compare the prognostic utility of UICC AS and AJCC PS in patients with high-risk luminal breast cancer.

**Methods:**

This retrospective study included patients who underwent surgery for ER-positive, HER2-negative breast cancer at Tokyo Metropolitan Komagome Hospital from 2011 to 2018. High-risk classification was defined based on MonarchE trial criteria: axillary lymph node metastasis, tumor diameter ≥5 cm, HG3, or Ki-67 ≥20%. Staging was performed using both UICC AS and AJCC PS, and prognostic values were evaluated via Kaplan-Meier survival analysis.

**Results:**

Among 105 eligible cases, 43 (41%) were downstaged when reclassified from UICC AS to AJCC PS. While UICC AS failed to yield significant stratification in terms of invasive disease-free survival (IDFS) and distant recurrence-free survival (DRFS), AJCC PS demonstrated significant prognostic discrimination (IDFS p = 0.014; DRFS p = 0.004).

**Conclusions:**

American Joint Committee on Cancer PS offers superior prognostic stratification compared to UICC AS in high-risk luminal breast cancer. Its incorporation into clinical practice may enable more personalized treatment approaches, particularly when identifying candidates for adjuvant abemaciclib therapy. Further validation through larger, prospective studies is warranted.

## Introduction

In 2020, approximately 2.2 million individuals were diagnosed with breast cancer globally, making it the most common malignancy among women ^(1)^. In the same year, over 680,000 deaths were attributed to breast cancer, ranking it as the fifth leading cause of cancer-related mortality worldwide ^(2)^.

The Tumor, Node, Metastasis (TNM) classification by the Union for International Cancer Control (UICC) is widely utilized for staging solid tumors, focusing on tumor size, regional lymph node involvement, and distant metastasis. Known as the anatomical stage (AS), this system provides a structural framework for evaluating disease severity and estimating prognosis ^(3)^. However, the heterogeneity of breast cancer limits the predictive utility of AS alone ^(4)^. Subtype-specific variations in treatment response and outcome underscore the need for biologically informed staging approaches.

Recognizing this need, the 8th edition of the American Joint Committee on Cancer (AJCC) introduced the Prognostic Stage (PS) in its staging manual. This revised system incorporates biological markers―estrogen receptor (ER), progesterone receptor (PgR), human epidermal growth factor receptor 2 (HER2), and histological grade (HG) into the traditional TNM framework to enhance prognostic precision and guide therapy ^(5)^.

Several studies have validated the prognostic relevance of AJCC PS across diverse breast cancer populations ^(6), (7), (8), (9)^. However, to our knowledge, no prior research has focused exclusively on high-risk luminal breast cancer patients, particularly those eligible for abemaciclib therapy as defined by the MonarchE trial criteria.

The MonarchE trial identified high-risk patients as those with either ≥4 axillary lymph node metastases or 1-3 positive nodes accompanied by at least one additional risk factor: tumor diameter ≥5 cm, HG3, or Ki-67 ≥20%. The MonarchE criteria are widely recognized as an international standard for defining high-risk hormone receptor-positive, HER2-negative early breast cancer patients who are eligible for adjuvant abemaciclib. The trial demonstrated significant improvement in invasive disease-free survival (IDFS) and distant recurrence-free survival (DRFS) with the addition of abemaciclib to standard endocrine therapy ^(10), (11), (12)^. Notably, staging in the MonarchE trial was based on UICC AS, without incorporating AJCC PS.

Given the increasing clinical relevance of biologically based staging, this study aimed to reassess prognosis in real-world MonarchE-eligible patients using AJCC PS in comparison with UICC AS.

## Materials and Methods

### Patients

This retrospective study included patients with ER-positive, HER2-negative breast cancer who underwent surgical treatment at Tokyo Metropolitan Komagome Hospital between January 2011 and December 2018. High-risk disease was defined according to the MonarchE criteria, which are internationally accepted for identifying patients eligible for adjuvant abemaciclib. Eligibility was determined based on MonarchE trial criteria, including either ≥4 axillary lymph node metastases, or 1-3 positive nodes along with at least one of the following features: invasive tumor diameter of ≥5 cm, HG 3, or Ki67 ≥20%. Patients who received neoadjuvant chemotherapy or endocrine therapy were excluded to ensure accurate pre-treatment staging. Postoperative radiation and chemotherapy were permitted. Patients with inflammatory breast cancer, deep vein thrombosis, or occult breast cancer were excluded, following MonarchE trial parameters.

### Data collection

Clinical and pathological data were retrospectively extracted from medical records. Tumor staging was assigned using both UICC AS and AJCC PS based on the 8th edition of their respective staging manuals. Comparative staging results were summarized in Table 1.

**Table 1.** Patients’ Characteristics.

	n＝105	%
Sex		
Female	105	100%
Male	0	0%
Mean age, (range), years old	54 (32-87)	
Mean observation period, (range) months	59.3 (9.7 -119)	
Menopausal		
Premenopausal	52	50%
Postmenopausal	53	50%
Procedure (breast)		
Mastectomy	88	84%
Breast conserving surgery	17	16%
Procedure (axillary lymph node)		
SNB	12	11%
SNB→Ax	32	30%
Ax	61	58%
Adjuvant chemotherapy		
Yes	80	76%
No	25	24%
Endocrine therapy		
Yes	104	99%
Aromatase inhibitor	56	61%
Tamoxifen	48	39%
No	1	1%
Radiation therapy		
Residual breast irradiation	16	15%
Postmastectomy radiation therapy	43	41%
No done	46	44%

Ax: axillary lymph node dissection; SNB: sentinel lymph node biopsy.

### Histological evaluation

Pathological diagnoses were performed by board-certified pathologists. HG was determined using a modified Nottingham Scarff-Bloom-Richardson scoring system ^(13)^. ER and PgR expression were evaluated by immunohistochemistry, with a threshold of >10% ^(14)^. Ki-67 immunohistochemical evaluation was performed according to standardized institutional procedures. Hotspot areas with the highest labeling index were selected, and at least two physicians, including one board-certified pathologist in Japan, independently assessed Ki-67. In cases of discrepant evaluation, a consensus was reached through joint review.

### Statistical analysis

Statistical analysis was performed using EZR software (EZR version 1.61) ^(15)^. A p-value of <0.05 was considered statistically significant. Kaplan-Meier survival curves were generated for IDFS, DRFS, and overall survival (OS). Staging groups were consolidated as follows: Stage I (Stage IA and IB), Stage II (IIA and IIB), and Stage III (IIIA, IIIB, and IIIC).

### Ethical approval and consent to participation

This study adhered to the principles outlined in the Declaration of Helsinki and was conducted in accordance with the Japanese Clinical Research Act and relevant enforcement ordinances. The study protocol received approval from the hospital’s Ethics Review Committee. All patients provided informed consent for the use of their clinical data in research.

## Results

**Figure 1.**
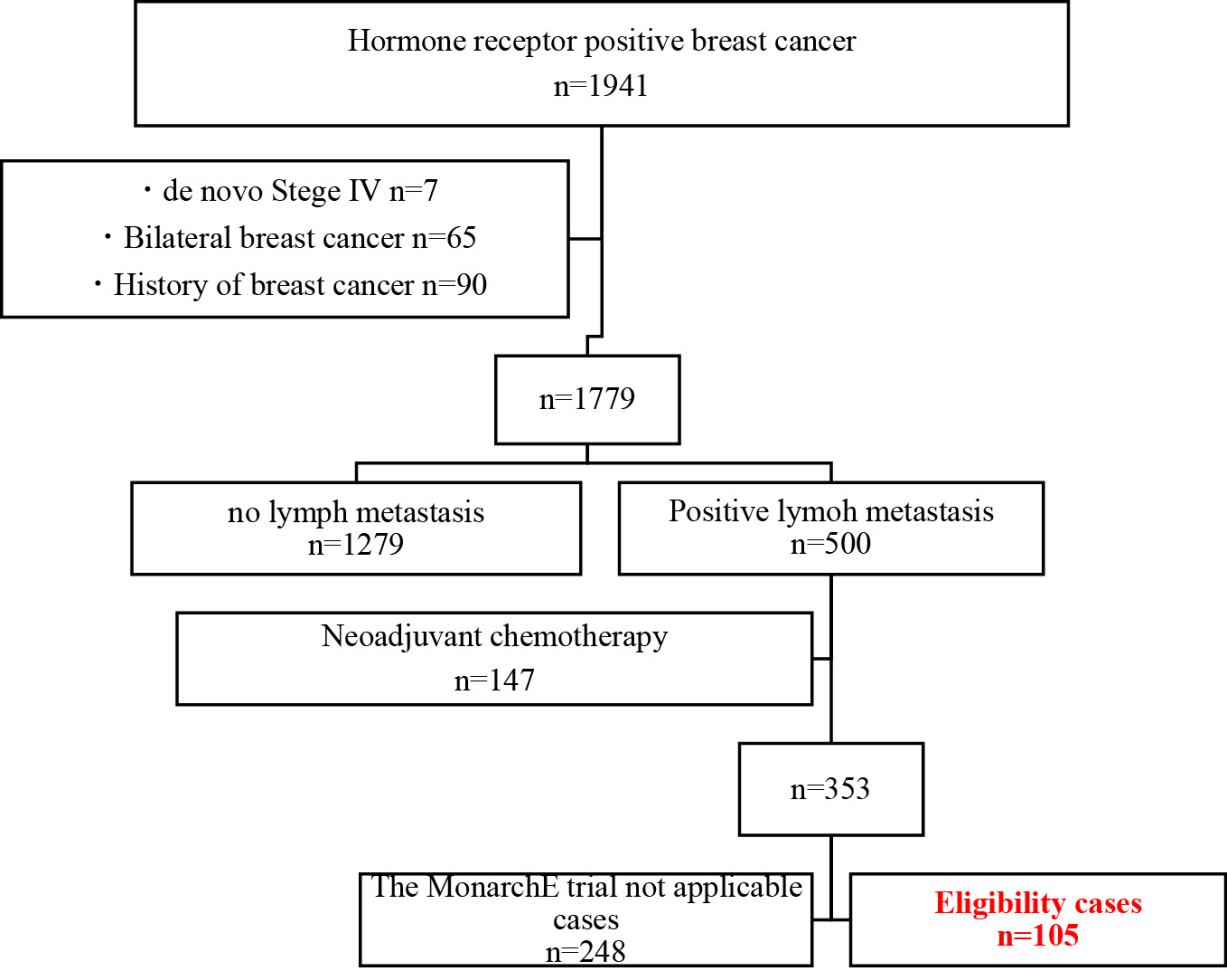
Consort diagram. During the study period, a total of 1,941 cases of luminal breast cancer were diagnosed at Komagome Hospital. Among them, 1,779 patients underwent surgery, and 500 had lymph node metastases. After excluding 147 patients who received preoperative chemotherapy, 353 cases remained, of which 105 met the eligibility criteria for the MonarchE trial. These findings provide a real-world perspective on patient selection for adjuvant therapy and may contribute to optimizing treatment strategies for high-risk luminal breast cancer.

A total of 1,941 patients were diagnosed with luminal breast cancer during the study period. Among them, 1,779 underwent surgical resection, and 500 had confirmed axillary lymph node metastases. After excluding 147 patients who had received neoadjuvant chemotherapy, 353 cases remained, of which 105 met the MonarchE eligibility criteria (Figure 1).

The median age was 54 years (range: 32-87), and the median follow-up period was 59.3 months (range: 9.7-119). Postoperative adjuvant chemotherapy and endocrine therapy were administered in 76% and 99% of cases, respectively (Table 1). The median tumor size was 26 mm (range: 6-256 mm), with 25% of tumors classified as T3 (≥5 cm). Additionally, 38% of patients had ≥4 axillary lymph node metastases, 68% had HG3 tumors, and 58% showed Ki-67 ≥20% (Table 2).

**Table 2.** Pathological Examination.

	n＝105	%
Median tumor size (range), mm	26 (6-25)	
T		
T1 (～2 cm)	32	30%
T2 (2～5 cm)	47	45%
T3 (5 cm～)	25	22%
T4	1	1%
N		
N1mic	8	8%
N1	55	52%
N2	23	21%
N3	19	18%
Number of lymph nodes metastasis		
1～3	65	62%
≥4	40	38%
Histological type		
Invasive ductal carcinoma	100	95%
Mucinous carcinoma	4	4%
Invasive lobular carcinoma	1	1%
Histological grade		
1	11	11%
2	24	29%
3	70	70%
Estrogen receptor		
≥50%	97	93%
10～50%	8	8%
1～9%	0	0%
<1%	0	0%
Progesterone receptor		
≥50%	50	48%
10～50%	31	30%
1～9%	13	12%
<1%	11	10%
Ki67		
<20%	22	21%
≥20%	75	58%
Unknown	8	8%

A pathological comparison between UICC AS Stage III and AJCC PS Stage III was performed in Table S1. Patients classified as AJCC PS Stage III had a higher nodal burden and a higher proportion of PgR-negative tumors than those classified as UICC AS Stage III. Figure S1 describes the reasons for downstaging. The most common reason was automatic downstaging due to luminal subtype, observed in 44 cases (54%), followed by tumor grade, which accounted for 29 cases (36%).

**Figure 2.**
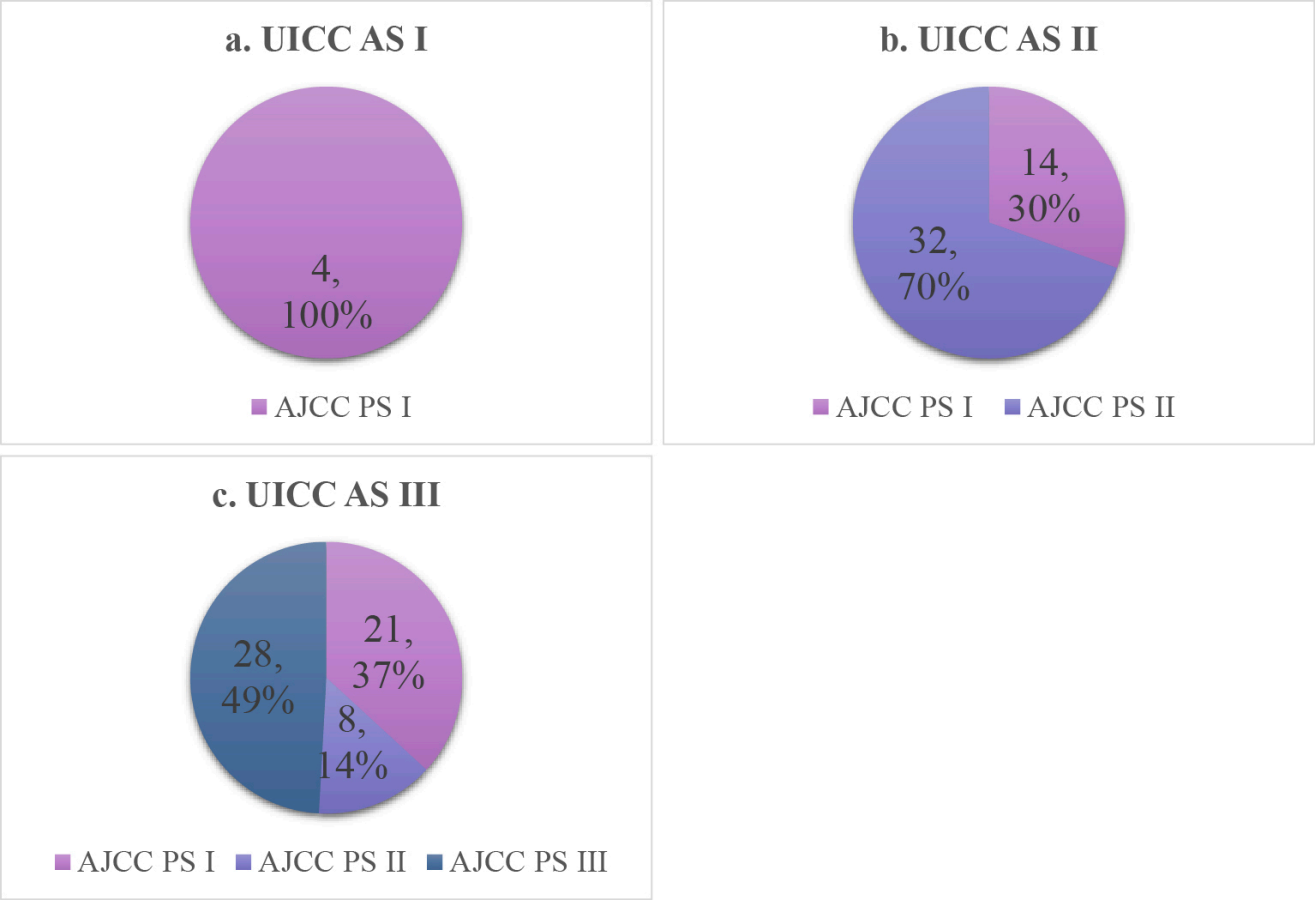
Comparison of UICC AS and the AJCC PS. In UICC AS I, all cases were classified as AJCC PS I. In UICC AS II, 14 cases (32%) were downstaged to AJCC PS I. In UICC AS III, 21 cases (37%) were downstaged to AJCC PS II, and 8 cases (14%) were downstaged to AJCC PS I. AJCC: American Joint Committee on Cancer; AS: anatomical stage; PS: prognostic stage; UICC: Union for International Cancer Control.

Reclassification from UICC AS to AJCC PS led to downstaging in 43 patients (41%). Specifically, Stage IA increased from 0 cases to 18 cases, Stage IB from 4 cases to 21 cases, while Stage IIIA and IIIC decreased from 38 to 13 and from 19 to 2 cases, respectively (Table 3, Figure 2).

**Table 3.** Comparison of Numbers and Percentages of AS and PS.

	AS	%	PS	%
StageⅠA	0	0	18	17
StageⅠB	4	4	21	20
StageⅡA	19	18	24	23
StageⅡB	25	23	14	13
StageⅢA	38	36	13	12
StageⅢB	0	0	13	12
StageⅢC	19	18	2	2

AS: anatomical stage; PS: prognostic stage.

**Figure 3.**
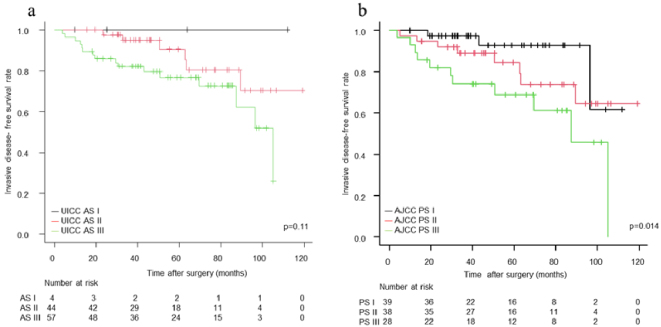
Comparison of invasive disease-free survival rate. (a) UICC AS. (b) AJCC PS. (a) IDFS was analyzed according to stages I, II, and III in UICC AS. Although the incidence of IDFS events was higher in stages II and III, no significant difference was observed among the three groups (p = 0.11). The five-year IDFS event rates were 100% for UICC AS I, 90.4% for UICC AS II, and 76.7% for UICC AS III. (b) IDFS was analyzed according to stages I, II, and III in AJCC PS, revealing a significant difference among the three groups (p = 0.014). The five-year IDFS event rates were 92.7% for AJCC PS I, 84.4% for AJCC PS II, and 68.9% for AJCC PS III. AJCC: American Joint Committee on Cancer; AS: anatomical stage; IDFS: invasive disease-free survival; PS: prognostic stage; UICC: Union for International Cancer Control.

**Figure 4.**
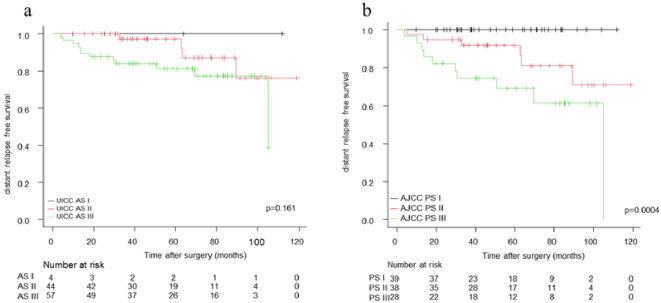
Comparison of DRFS rate (a) AS. (b) AJCC PS. (a) DRFS was analyzed according to stages I, II, and III in UICC AS. Although the incidence of IDFS events was higher in stages II and III, no significant difference was observed among the three groups (p = 0.161). The five-year IDFS event rates were 100% for UICC AS I, 97.3% for UICC AS II, and 81.1% for UICC AS III. (b) DRFS was analyzed according to stages I, II, and III in AJCC PS, revealing a significant difference among the three groups (p = 0.004). The five-year IDFS event rates were 100% for AJCC PS I, 91.8% for AJCC PS II, and 69.0% for AJCC PS III. AJCC: American Joint Committee on Cancer; AS: anatomical stage; DRFS: distant recurrence-free survival; IDFS: invasive disease-free survival; PS: prognostic stage; UICC: Union for International Cancer Control.

**Figure 5.**
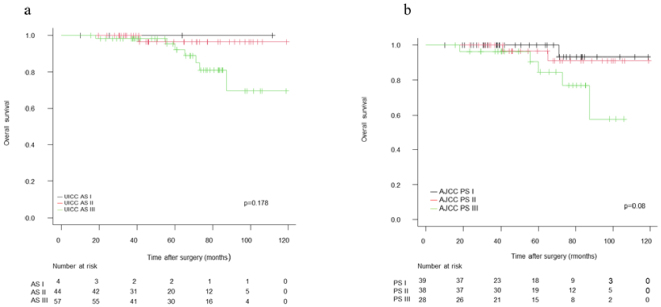
Comparison of OS rate (a) UICC AS. (b) AJCC PS). (a) OS was analyzed according to stages I, II, and III in UICC AS. Although the incidence of OS events was higher in stages II and III, no significant difference was observed among the three groups (p = 0.178). The five-year OS event rates were 100% for UICC AS I, 96.7% for UICC AS II, and 92.1% for UICC AS III. (b) OS was analyzed according to stages I, II, and III in AJCC PS, revealing no significant difference among the three groups (p = 0.08). The five-year OS event rates were 100% for AJCC PS I, 96.4% for AJCC PS II, and 84.6% for AJCC PS III. AJCC: American Joint Committee on Cancer; AS: anatomical stage; OS: overall survival; PS: prognostic stage; UICC: Union for International Cancer Control.

Kaplan-Meier survival analyses demonstrated that IDFS and DRFS were not significantly stratified by UICC AS (IDFS p = 0.11; DRFS p = 0.161). In contrast, AJCC PS provided significant prognosis discrimination (IDFS p = 0.014; DRFS p = 0.004) (Figures 3 and 4). No statistically significant differences in OS were observed between the two staging systems (Figure 5).

## Discussion

This study highlights the prognostic advantages of AJCC PS over UICC AS in patients with high-risk luminal breast cancer, particularly those who meet the MonarchE trial criteria. Unlike the MonarchE trial, our analysis excluded patients receiving neoadjuvant chemotherapy to preserve the accuracy of pre-treatment staging. Notably, 41% of cases were downstaged under AJCC PS.

Our results indicate that AJCC PS offers clearer prognostic stratification for IDFS and DRFS. The five-year IDFS for AJCC PS Stage I was 92.7%, whereas UICC AS Stage I had a rate of 100%, reflecting local recurrence without distant metastasis or second malignancies. These findings suggest that patients classified as Stage I by AJCC PS may achieve favorable outcomes without abemaciclib.

To better understand the differences between AS and PS within Stage III disease, we performed a comparative analysis. A greater number of positive lymph nodes leads to classification as Stage IIIC, which remains Stage III even after reclassification from AS to PS. In contrast, tumors classified as AS Stage IIIA or IIIB with PgR-positive status are downstaged to Stage IIB according to the PS system. Consequently, AJCC PS Stage III identifies a biologically aggressive subgroup characterized by a higher number of positive lymph nodes and/or PgR-negative disease, which is associated with poorer prognosis.

Reclassification using AJCC PS reduced the number of patients classified as Stage III from 57 to 28. Furthermore, within the AJCC PS-defined Stage III group, DRFS was worse than in the corresponding UICC AS-defined Stage III group, suggesting that AJCC PS-based staging enriches for a subgroup with more aggressive disease. This finding indicates the potential of AJCC PS to better identify patients who may derive the greatest benefit from adjuvant abemaciclib therapy.

Despite the observed advantages in IDFS and DRFS, OS did not significantly differ between staging systems, which may be attributable to the inherently favorable prognosis of luminal breast cancer. Prior studies, Tanaka et al. and Abdel-Rahman et al. ^(6), (7)^, have similarly demonstrated the superior prognostic relevance of AJCC PS compared to UICC PS ^(6), (7), (16)^.

Given that abemaciclib is associated with adverse events such as neutropenia, thromboembolism, and interstitial lung disease ^(17)^, its use should be carefully considered. AJCC PS may serve as a valuable tool to better individualize treatment strategies, especially when determining the need for adjuvant abemaciclib.

Limitations of this study include its single-center, retrospective design, limited sample size, and lack of information on chemotherapy dose intensity. Furthermore, many patients were treated before Oncotype DX testing became widely available in Japan, potentially influencing treatment decisions. The relatively short follow-up period may also underrepresent the incidence of late recurrence, which is characteristic of luminal subtypes ^(18)^. Another limitation of this study is the relatively short median follow-up period of 59.3 months. Given that luminal breast cancer is characterized by late recurrences, this follow-up duration may not have been sufficient to fully capture long-term recurrence patterns and survival outcomes. With a longer observation period, differences in OS might have emerged.

These limitations notwithstanding, our findings support the integration of AJCC PS into routine clinical practice to enhance prognostic assessment and guide more tailored treatment planning for high-risk luminal breast cancer.

### Conclusions

AJCC PS provides superior prognostic stratification compared to UICC AS in patients with high-risk luminal breast cancer. PS not only downstaged a substantial number of patients but also identified a subgroup with poorer DRFS within Stage III, suggesting its utility in selecting candidates who may benefit most from adjuvant abemaciclib. Incorporating PS into clinical practice may enhance personalized treatment planning in this population.

## Article Information

### Author Contributions

All authors contributed to the study conception and design. Material preparation, data collection, and analysis were performed by Mio Adachi. The first draft of the manuscript was written by Toshiyuki Ishiba, Takashi Kuwayama, and Tomoyuki Aruga, and all authors commented on previous versions of the manuscript. All authors read and approved the final manuscript.

### Conflicts of Interest

Tomoyuki Aruga has received honoraria from Pfizer, Eli Lilly, AstraZeneca, NSD, and Daiichi Sankyo and research funding from Pfizer.

### Approval by Institutional Review Board (IRB)

This study was conducted in accordance with the Declaration of Helsinki and approved by the Institutional Review Board of Tokyo Metropolitan Cancer and Infectious Disease Center Komagome Hospital (protocol no.: 2,971; date: October 11, 2022).

## Supplement

Supplementary Material

## References

[1] Weiss A, Chavez-MacGregor M, Lichtensztajn DY, et al. Validation study of the American Joint Committee on Cancer eighth edition prognostic stage compared with the anatomic stage in breast cancer. JAMA Oncol. 2018;4(2):203-9. 29222540 10.1001/jamaoncol.2017.4298 PMC5838705

[2] Abdoli G, Bottai M, Sandelin K, et al. Breast cancer diagnosis and mortality by tumor stage and migration background in a nationwide cohort study in Sweden. Breast. 2017;31:57-65. 27810701 10.1016/j.breast.2016.10.004

[3] Wang M, Chen H, Wu K, et al. Evaluation of the prognostic stage in the 8th edition of the American Joint Committee on Cancer in locally advanced breast cancer: an analysis based on SEER 18 database. Breast. 2018;37:56-63. 29100045 10.1016/j.breast.2017.10.011

[4] Amin MB, Greene FL, Edge SB, et al. The eighth edition AJCC cancer staging manual: continuing to build a bridge from a population-based to a more “personalized” approach to cancer staging. CA Cancer J Clin. 2017;67(2):93-9. 28094848 10.3322/caac.21388

[5] Goldhirsch A, Wood WC, Gelber RD, et al. Progress and promise: highlights of the international expert consensus on the primary therapy of early breast cancer 2007. Ann Oncol. 2007;18(7):1133-44. 17675394 10.1093/annonc/mdm271

[6] Tanaka R, Yamagishi Y, 1 3, Koiwai T et al. Comparison between AJCC 8th prognostic stage and UICC anatomical stage in patients with primary breast cancer: a single institutional retrospective study. Breast Cancer. 2020;27:1114-25. 32495291 10.1007/s12282-020-01115-x PMC7567685

[7] Abdel-Rahman O. Validation of the 8th AJCC prognostic staging system for breast cancer in a population-based setting. Breast Cancer Res Treat. 2018;168(1):269-75. 29143220 10.1007/s10549-017-4577-x

[8] Sung H, Ferlay J, Siegel RL, et al. Global cancer statistics 2020: GLOBOCAN estimates of incidence and mortality worldwide for 36 cancers in 185 countries. CA Cancer J Clin. 2021;71(3):209-49. 33538338 10.3322/caac.21660

[9] Dieci MV, Bisagni G, Brandes AA, et al. Validation of the AJCC prognostic stage for HER2-positive breast cancer in the ShortHER trial. BMC Med. 2019;17(1):207. 31747948 10.1186/s12916-019-1445-z PMC6868696

[10] Johnston SRD, Harbeck N, Hegg R, et al. Abemaciclib combined with endocrine therapy for the adjuvant treatment of HR+, HER2-, node-positive, high-risk, early breast cancer (monarchE). J Clin Oncol. 2020;38(34):3987-98. 32954927 10.1200/JCO.20.02514 PMC7768339

[11] Martin M, Hegg R, Kim SB, et al. Treatment with adjuvant abemaciclib plus endocrine therapy in patients with high-risk early breast cancer who received neoadjuvant chemotherapy: a prespecified analysis of the monarchE randomized clinical trial. JAMA Oncol. 2022;8(8):1190-4. 35653145 10.1001/jamaoncol.2022.1488 PMC9164117

[12] Johnston SRD, Toi M, O’Shaughnessy J, et al. Abemaciclib plus endocrine therapy for hormone receptor-positive, HER2-negative, node-positive, high-risk early breast cancer (monarchE): results from a preplanned interim analysis of a randomised, open-label, phase 3 trial. Lancet Oncol. 2023;24(1):77-90. 36493792 10.1016/S1470-2045(22)00694-5 PMC11200328

[13] Elston EW, Ellis IO. Method for grading breast cancer. J Clin Pathol. 1993;46(2):189-90. 10.1136/jcp.46.2.189-b PMC501162 8459046

[14] Hammond MEH, Hayes DF, Dowsett M, et al. American Society of Clinical Oncology/College of American Pathologists guideline recommendations for immunohistochemical testing of estrogen and progesterone receptors in breast cancer. J Clin Oncol. 2010;28(16):2784-95. 20404251 10.1200/JCO.2009.25.6529 PMC2881855

[15] Kanda Y. Investigation of the freely available easy-to-use software ‘EZR’ for medical statistics. Bone Marrow Transplant. 2013;48(3):452-8. 23208313 10.1038/bmt.2012.244 PMC3590441

[16] Li X, Zhang Y, Meisel J, et al. Validation of the newly proposed American Joint Committee on Cancer (AJCC) breast cancer prognostic staging group and proposing a new staging system using the National Cancer Database. Breast Cancer Res Treat. 2018;171(2):303-13. 29948405 10.1007/s10549-018-4832-9

[17] Rugo HS, O’Shaughnessy J, Boyle F, et al. Adjuvant abemaciclib combined with endocrine therapy for high-risk early breast cancer: safety and patient-reported outcomes from the monarchE study. Ann Oncol. 2022;33(6):616-27. 35337972 10.1016/j.annonc.2022.03.006

[18] Salvo EM, Ramirez AO, Cueto J, et al. Risk of recurrence among patients with HR-positive, HER2-negative, early breast cancer receiving adjuvant endocrine therapy: a systematic review and meta-analysis. Breast. 2021;57:5-17. 33677313 10.1016/j.breast.2021.02.009 PMC8089079

