# Supplementary material for: Usefulness of Prognostic Stage in High-Risk Early Luminal Breast Cancer
Source: JMA J. 2026 Jul 10;9(4):900–8. doi: 10.31662/jmaj.2025-0485 (PMC13453904; doi:10.31662/jmaj.2025-0485)

Supplementary Table 1. Analysis of AS Stage III and PS Stage III.

	AS (n= 57) (%)	PS (n= 28)	P value
Tumor size, mean±SD, mm	51.6±38.7	57.1±47.6	0.57
Number of positive lymph nodes, mean ± SD	9.1±8.9	13.6±10.6	0.03
1～3<br>≧4	16 (28%)<br>41 (72%)	7 (25%)<br>21 (75%)	
Ki67<br>< 20%<br>≧ 20%<br>Unknown	 17 (30%)<br>37 (65%)<br>3 (5%)	 18 (64%)<br>10 (36%)<br>0 (0%)	Ki67 <20% vs ≧ 20% 0.005
Histological Grade<br>1 and 2<br>3	 35 (61%)<br>22 (39%)	 14 (50%)<br>14 (50%)	0.356
PgR<br>Positive<br>Negative	 35 (61%)<br>22 (39%)	 10 (36%)<br>18 (64%)	0.03
AS; anatomical stage, PS; prognostic stage, SD; standard deviation, PgR; Progesterone Receptor			

Supplementary Figure 1. Reason for Down Staging.

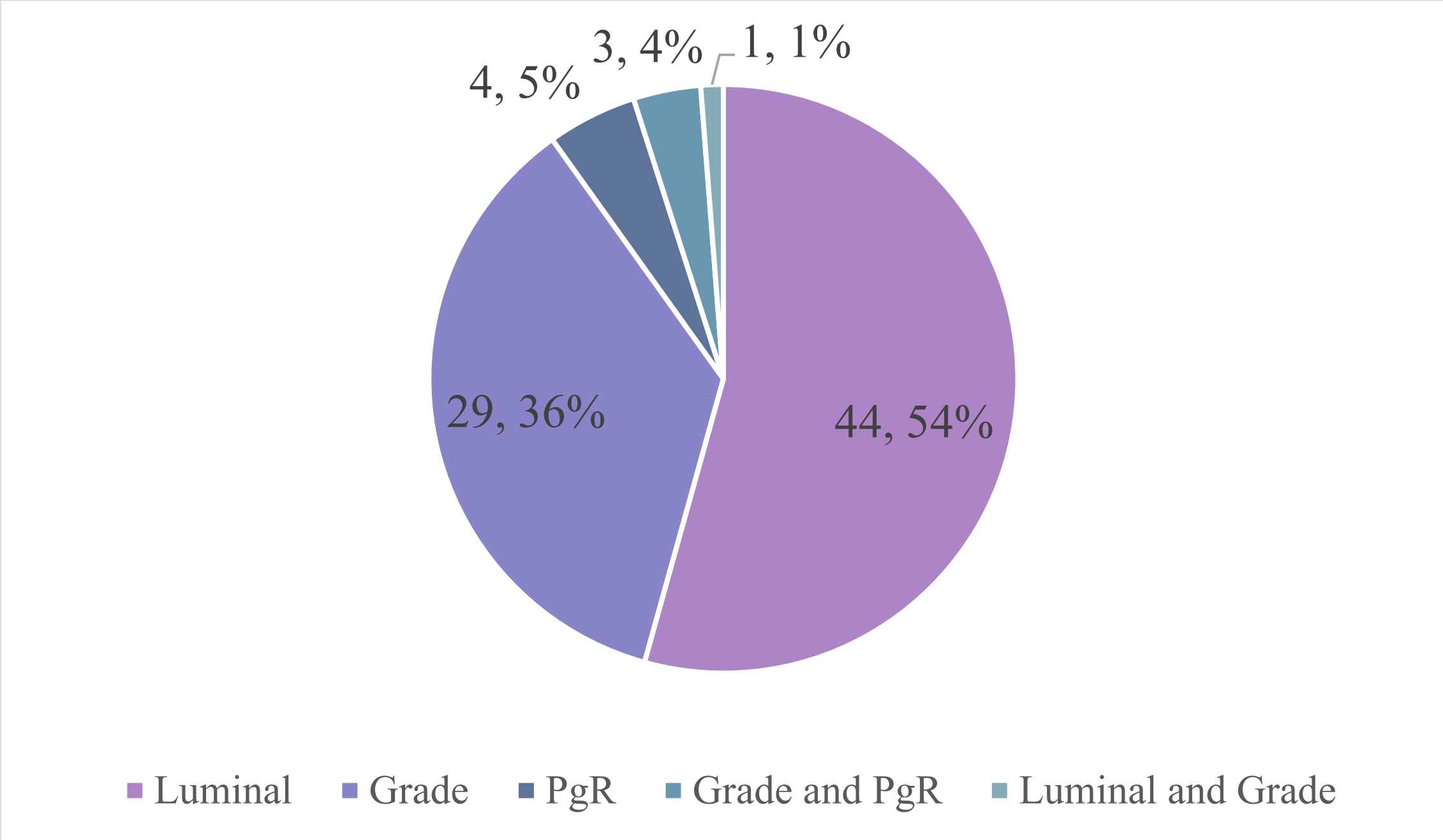

Supplement: Supplementary file 1 [file 2433-3298-9-4-0900-s001.pdf]
